# Sex Differences in the Anxiolytic Properties of Common Cannabis Terpenes, Linalool and β-Myrcene, in Mice

**DOI:** 10.3390/neurosci5040045

**Published:** 2024-12-03

**Authors:** Jasmin K. Wagner, Ella Gambell, Tucker Gibbons, Thomas J. Martin, Joshua S. Kaplan

**Affiliations:** 1 Behavioral Neuroscience Program, Psychology Department, Western Washington University, 516 High Street, Bellingham, WA 98229, USA; 2 Department of Research and Development, Abstrax Tech, 2661 Dow Avenue, Tustin, CA 92780, USA

**Keywords:** terpenes, cannabidiol, cannabis, monoterpenes, anxiety, linalool, myrcene, entourage effect

## Abstract

Volatile organic compounds, colloquially referred to as “terpenes”, have been proposed to impact the therapeutic qualities that are traditionally ascribed to cannabis. However, the contribution of these terpenes in anxiety, at relevant levels and exposure methods common with cannabis use, is lacking empirical assessment. We tested the anxiolytic properties of two prominent cannabis terpenes, linalool and β-myrcene, in male and female mice using short duration vapor pulls to model human inhalation when combusting flower or vaping cannabis oil. We observed sex differences in the locomotor effects in the open field and anxiolytic properties in the elevated plus maze of these terpenes that depended on their exposure characteristics. Both linalool and β-myrcene had anxiolytic effects in female mice when delivered in discrete vapor pulls over the course of 30 min. In male mice, only a single vapor hit containing linalool or β-myrcene had anxiolytic effects. The combination of sub-effective levels of linalool and the phytocannabinoid, cannabidiol (CBD), had synergistic anxiolytic effects in females, but these entourage effects between CBD and terpenes were absent with β-myrcene for females and for either terpene in males. Together, our findings reveal sex differences in the anxiolytic properties of common cannabis terpenes and highlight the potential benefits of unique combinations of CBD and terpenes in expanding the therapeutic dose window.

## 1. Introduction

Anxiety disorder is one of the most common mental illnesses [1] and is becoming more prevalent [2]. Self-medication for anxiety disorders with *Cannabis sativa* L. [3,4,5,6,7] is increasingly common due to incomplete efficacy and moderate side effect profiles with current prescription medications [8,9]. Anxiety-related health complications (e.g., poor sleep) are often reported to be one of the most common reasons for off-label medicinal cannabis use [10,11], necessitating further empirical investigation into the potential therapeutic efficacy of cannabis in the treatment of anxiety. However, the diversity of phytochemicals produced by cannabis is immensely complex [12], and it is this variability in phytochemical composition that contributes to the unique effects and varying levels of efficacy conferred by particular strains and products [13,14]. Understanding how these different chemicals impact anxiety alone and in combination could lead to improved cannabis-based therapeutic strategies for mitigating stress and anxiety. 

Over 200 volatile organic compounds can be produced by *Cannabis sativa* L. and fall into numerous categories based on their chemical structure. One abundant category is colloquially known as “terpenes”, which includes several subcategories, thus illustrating the immense diversity of volatile organic compounds, and in particular, terpene profiles [12,15]. Historically, the therapeutic investigation of terpenes stemmed from studying essential oils of plants used in aromatherapy [16,17], and many of the claims of therapeutic efficacy of monoterpenes were derived from these oils which often contain multiple terpenes. These essential oils have been demonstrated to reduce stress [18], reduce anxiety [19], and improve mood [20,21]. The effects of monoterpenes, independently, are understudied. We focused our study here on two common and abundant terpenes in cannabis flower and commercial products, β-myrcene and linalool [22], that have purported anxiolytic qualities [17,23]. 

β-myrcene has been used in traditional medicine approaches for its sedating and anxiolytic properties [24]. It is a dominant monoterpene found in numerous plants including hops, some citrus (e.g., mangos), and lemongrass [23]. Despite its historical inclusion in folk remedies, there is sparse evidence of β-myrcene’s anxiolytic and sedating effects as a monoterpene [25], in relevant doses found in cannabis and cannabis products, or inhaled to more closely model the pharmacokinetic properties of common inhalation or tincture methods of cannabis consumption [26].

Linalool is the dominant monoterpenoid in lavender essential oil and has anxiolytic [27,28] and sedating properties [29,30] in pre-clinical rodent models. Notably, the relevant linalool inhalation experiments almost exclusively use male animals and a constant exposure paradigm that more closely mimics aromatherapy than the short discrete exposure periods from puff patterns when combusting or vaporizing cannabinoids. 

Cannabidiol (CBD) is a non-intoxicating phytocannabinoid produced by cannabis and is dominant in the hemp variety, which is typically classified as having less than 0.3% Δ^9^-THC [31]. CBD has been extensively studied for its anxiolytic properties [13,32], which have revealed that these anxiolytic effects are only achieved within a narrow dose–efficacy window [33,34,35,36]. This narrow dosing window may be difficult for people to achieve consistently, if at all, and perhaps has contributed to mixed results of CBD’s anxiolytic efficacy in human trials [37]. Expanding this dose–efficacy window would lead to improved cannabis-based product development and reliable therapeutic utility for those seeking relief from anxiety and anxiety-related complications. 

Although CBD is often studied in rodent and human laboratory experiments as a purified isolate, CBD is just one of hundreds of pharmacodynamically active chemicals produced by cannabis [13,38]. Unique blends of volatile organic compounds produced by the cannabis plant contribute to specific odors and flavors of different varietals. Many are also pharmacodynamically active and have been proposed to contribute to some of the unique therapeutic profiles of particular cannabis strains and products either by independent action or in synergistic interaction with phytocannabinoids such as CBD [8,12,14,31,39,40]. Therefore, cannabis flower and whole-plant oil extracts available in medicinal and recreational markets may confer different net effects than CBD isolates [14]. Understanding the impact of common volatile organic compounds in cannabis on the brain and behavior is important for improving the predictive validity of cannabis-based treatment approaches and enhancing their efficacy.

Together, the dearth of empirical evidence of these common cannabis terpenes in relevant concentrations, using relevant cannabis inhalation patterns and across both sexes, coupled to the increasing popularity of CBD-rich products for off-label self-medication for anxiety treatment, reveals a need for further investigation into these terpenes on their own and in combination with CBD. The purpose of this investigation was to empirically test the anxiolytic properties of these two common cannabis terpenes alone and in combination with CBD in male and female mice using relevant vaporization patterns to mimic discrete inhalation events common in human cannabis smoking or vaping. We reveal different anxiolytic characteristics of these terpenes between male and female mice. 

## 2. Materials and Methods

### 2.1. Animals

For this research, 78 male and 86 female C57BL/6J mice (Jackson Laboratories, Bar Harbor, ME, USA) were bred in-house at Western Washington University and used in the experiments. Mice were housed in standard laboratory housing in groups of 3–5 mice per cage on a 12 h light/dark cycle (lights on at 0700) with food (Formula 5663, Mazuri Rat and Mouse Diet) and water provided ad libitum. A consistent experimenter handled and habituated the mice for a minimum of 5 min/day for 3 days prior to the experimental assessment. Testing and exposure rooms were 20 °C. All drug exposures and behavioral testing were performed during the light cycle. All procedures were approved by Western Washington University’s Institutional Animal Care and Use Committee and conform to the regulations detailed in the National Institutes of Health’s Guide for the Care and Use of Laboratory Animals. 

### 2.2. Drugs and Vape Oils

CBD isolate (>98% purity) and the volatile organic compounds (referred to as “terpenes”), β-myrcene and linalool, were gifted from Abstrax Tech (Tustin, CA, USA). CBD or the terpenes were dissolved in a vehicle solution comprising 70% vegetable glycerin and 30% propylene glycol purchased from La Jolla Alcohol Research, Inc. (La Jolla, CA, USA). The terpene and CBD concentrations in the vape oils were 5% and 30 mg/mL, respectively, which are common for commercially available products. Vape oils were prepared on the day of experiments and thoroughly mixed until no separation was observed by visual inspection. 

### 2.3. Drug Administration 

Terpene and CBD vapor was passively delivered to subjects across four 36 cm × 27 cm × 23 cm (L × W × H)~17 L passive vapor inhalation chambers (La Jolla Alcohol Research, Inc.). Unidirectional airflow was established by a vacuum pump that would pull air through the chambers at a rate of 7.5 L/min and monitored by a meter at the intake port. Vapor delivery was computer controlled and programmed to send a precise and discrete electrical current to the base of the atomizer, which would heat a commercial SMOK TFV8 Baby Beast Tank with a 0.4 Ω atomizer coil (40–60 W range) filled with freshly prepared vape oil each day of the experiments. Air was then pulled through the chamber and passed through an in-line Whatman HEPA-Cap filter (Millipore-Sigma, St. Louis, MI, USA). The air in the chambers appeared visibly clear of vapor prior to subsequent pull. Vapor pulls lasted for 6 s and were delivered every 5 min for 30 min (starting at time point 0 for a total of 7 pulls per session). One notable exception was the short exposure experiments during which mice only received a single 3 s pull at the end of the 30 min session. We had previously identified that each 6 s vapor pull exposes mice to vapor for approximately 2 min (120.25 ± 4.55 s) at progressively decreasing concentrations, as the air was replaced in the exposure chambers [41]. 

### 2.4. Behavioral Assessment

Behavioral assessment began between postnatal days 120 and 200. Animals were age matched within each experiment and resulting analysis. Exposure conditions were counterbalanced for all experiments. Terpene experiments were conducted semi-within subjects such that each animal was exposed to only the vehicle and a single terpene spaced at 2 weeks. The exception was that the entourage effect experiments were conducted as a between-subjects design to reduce practice effects by running animals on the elevated plus maze more than twice. After treatment vapor exposure, animals remained in the chambers for an additional 7 min following the last vapor exposure before being moved to the behavioral room. Animal behavior was tested approximately 15 min following the last vapor exposure. Animal movement was recorded in the presence of overhead fluorescent lighting using a digital camera (Microsoft LifeCam HD-3000) mounted above the behavioral apparatus. Behavior was analyzed using ezTrack open source animal tracking software [42]. Each video was checked for accurate assessment by visually inspecting output Bokeh plots and calculating total ratios to ensure that 100% of their behavior was captured in analysis. At the end of each trial, the behavioral apparatus was cleaned with 70% ethanol and wiped with paper towels. Both males and females were tested on the same apparatus.

### 2.5. Open Field Test

The open field arena is made of white plexiglass (44 × 44 cm). The center quadrant is a 22 × 22 cm square centered 11 cm from each wall created by the ezTrack software to measure time spent in the center of the chamber. Each test began by placing the subject near the same wall of the arena and allowing exploration for 10 min. Total distance traveled and time in the center quadrant were the primary dependent variables. Experimenters left the behavioral room during the experiment and monitored behavior on a computer monitor through a narrow window. The open field test was conducted with full overhead lighting.

### 2.6. Elevated Plus Maze

The elevated plus maze (EPM) arena consists of 4 60 cm × 6 cm maze arms that are connected in the middle at a 6 × 6 cm open center (total 126 cm in length). Two “closed” arms are surrounded by 21 cm opaque plexiglass walls on 3 sides while the other two “open arms” are open on all sides. The maze is elevated 93 cm above the floor. The EPM was performed under full overhead lighting. To begin, subjects were placed in the center of the white plus-shaped maze after which they were allowed to explore for 5 min. The primary dependent variables were the ratio of time spent in the open arms/closed arms and the number of entries into the open arms. Experimenters left the behavioral room during the experiment and monitored behavior on a computer monitor through a narrow window. 

### 2.7. Olfactory Detection

These procedures were adapted from the cotton tip-based olfactory habituation test described in [43]. This test was conducted with each mouse placed individually in a standard holding cage with fresh bedding. A 6 inch cotton-tipped wooden applicator with one side wrapped in cotton was dipped into one of the 5 prepared solutions (vehicle; 0.5% linalool, 5% linalool, 0.5% β-myrcene, or 5% β-myrcene). Cotton applicators were placed into 15 mL conical tubes to prevent direct engagement with the applicator. During the test, the cotton tip was placed approximately 5 cm from the end walls in the middle of the cage and approximately 8 cm from the cage floor. Cumulative time sniffing the tip was recorded with a stopwatch during 4 1 min trials with 2 min inter-trial intervals. After these 4 trials, a new odorant condition was presented for an additional 4 trials until all conditions were completed. The order of solution presentation was counterbalanced across subjects. To assess reliable change between the early stages of the experiment and the latter, we averaged the sniff times in the last two trials and compared this to averaged sniff times in the first two trials.

### 2.8. Statistical Analysis

All data are shown as mean ± standard error of the mean (SEM) and analyzed by either two-way between-subjects ANOVA, two-way mixed measures ANOVA, or paired *t*-tests where appropriate, using Sigma Plot 14.0 software (SYSTAT Software) with an alpha set at 0.05. All tests are two-tailed. When appropriate, we used two-way ANOVA to assess the effect of sex and exposure condition on the dependent variables. Tukey’s HSD post hoc comparisons were used to analyze statistically significant main effects and interactions. Descriptive statistics (mean ± SEM) are included in Supplementary Table S1. For all figures, * Indicates *p* < 0.05; ** indicates *p* < 0.01; *** indicates *p* < 0.001. 

## 3. Results

### 3.1. Sex Differences in the Anxiolytic and Locomotor Effects of Linalool and β-Myrcene

**Figure 1:**
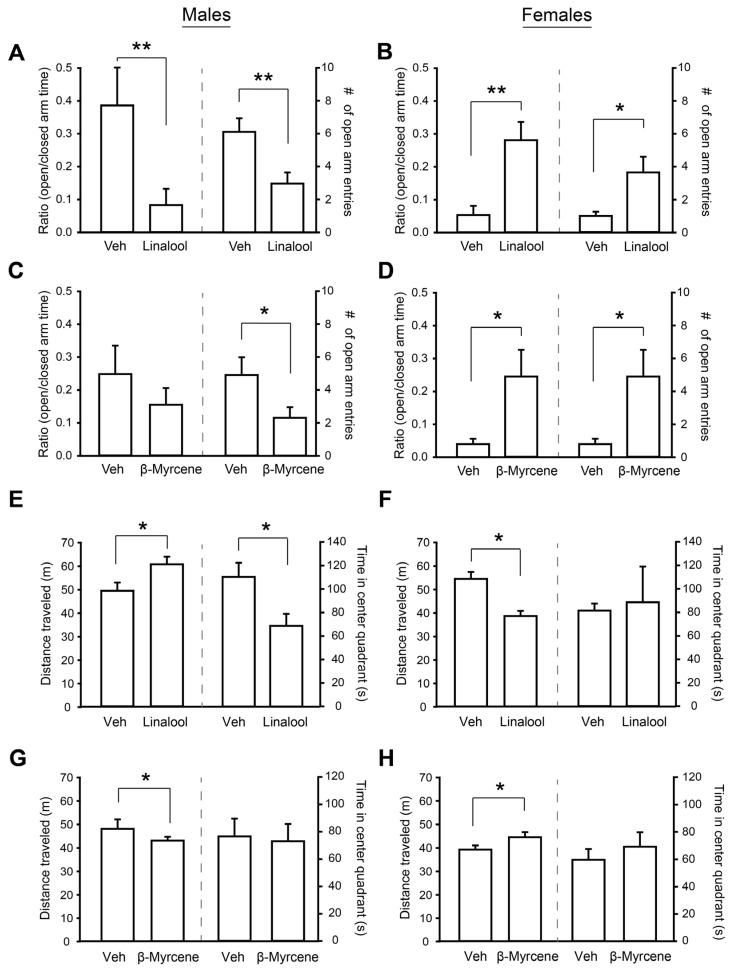
Sex differences in terpene effects on anxiety and locomotor activity. (**A**,**B**) Summary bar charts showing the ratios of time mice spent in the open relative to closed arms (left) and the numbers of open arm entries (right) in the elevated plus maze in males, n = 6 (**A**) and females, n = 6 (**B**) after 30 min of linalool vapor pulls. (**C**,**D**) Summary bar charts showing the ratios of time mice spent in the open relative to closed arms and the numbers of open arm entries in the elevated plus maze in males, n = 10 (**C**) and females, n = 7 (**D**) after 30 min of β-myrcene vapor pulls. (**E**,**F**) Summary bar charts showing the distances traveled (left) and times spent in the center quadrant (right) of the open field test box in males, n = 5 (**E**) and females, n = 5 (**F**) after 30 min of linalool vapor pulls. (**G**,**H**) Summary bar charts showing the distances traveled (left) and times spent in the center quadrant (right) of the open field test box in males, n = 9 (**G**) and females, n = 10 (**H**) after 30 min of linalool vapor pulls. Two-way mixed measures ANOVA assessed interactions between sex and treatment as well as any main effects. * indicates *p* = 0.05; ** *p* < 0.01. *p* values generated by Tukey’s HSD post hoc comparisons when appropriate. Data are presented as mean ± SEM.

We first sought to identify the impact of β-myrcene or linalool on anxiety-like and locomotor behavior using an acute vaporization exposure method where mice were exposed to 6 s vapor pulls every 5 min for 30 min of a mixture comprising 5% terpene and 95% vehicle. A 5% terpene concentration was chosen because it represents the upper end of naturally-occurring terpenes in cannabis [44], but levels can notably be much higher by adding exogenous terpenes to cannabis vape oils [45]. Anxiety-like behavior was assessed in both male and female mice using the well-validated EPM [46]. The ratio of time spent in the open relative to the closed arms and number of open arms entries was interpreted to be directly correlated with the terpene’s anxiolytic properties. Each terpene was tested independently against a vehicle exposure in a counterbalanced manner. A two-way mixed measures ANOVA identified a significant interaction between sex and linalool on the open/closed ratio, F(1,10) = 15.87, *p* = 0.003, and open arm entries, F(1,10) = 51.04, *p* = 0.002. We observed antipodal effects of linalool in male and female mice: linalool increased the open/closed ratio and number of open arm entries in female mice and reduced it in males (all *p* < 0.05; Figure 1A,B), consistent with reduced anxiolytic effects in females and anxiogenic effects in males. There was similarly a significant interaction between sex and β-myrcene on the open/closed ratio, F(1,15) = 5.82, *p* = 0.029, and open arm entries, F(1,15) = 97.62, *p* < 0.001. β-myrcene increased the open/closed ratio and number of open arm entries in females (*p* < 0.05; Figure 1C,D), but only decreased the number of open arm entries in males. Males had higher open/closed arm time ratios and open arm entries for the vehicle condition than females in both terpene experiments (all *p* < 0.05). However, the repeated measures design controlled for baseline differences to the vehicle and enabled us to detect terpene effects within each sex. We next tested the effect of the terpenes on locomotor activity in the open field. A two-way mixed measures ANOVA found a significant interaction between sex and linalool exposure, F(1,8) = 98.13, *p* < 0.001. This interaction was once again driven by linalool having antipodal effects in male and female mice: it reduced the distance traveled in females but enhanced it in males (all *p* < 0.05; Figure 1E,F). Similarly, a two-way mixed measures ANOVA found a significant interaction between sex and β-myrcene exposure, F(1,17) = 10.20, *p* = 0.005, caused by antipodal effects of β-myrcene between the sexes: β-myrcene increased the distance traveled in females but reduced it in males (all *p* < 0.05; Figure 1G,H). Linalool reduced the time in the center quadrant in males, but no other terpene effects were found on time spent in the center quadrant of the open field chamber. These findings suggest that linalool and β-myrcene have sex-specific impacts on anxiety and locomotor behavior, but there is no clear predictive relationship between terpene effects on locomotor activity and anxiety that accounts for behavior across both sexes. 

### 3.2. Male Mice Are More Sensitive to Repeated Terpene Exposures than Females

**Figure 2:**
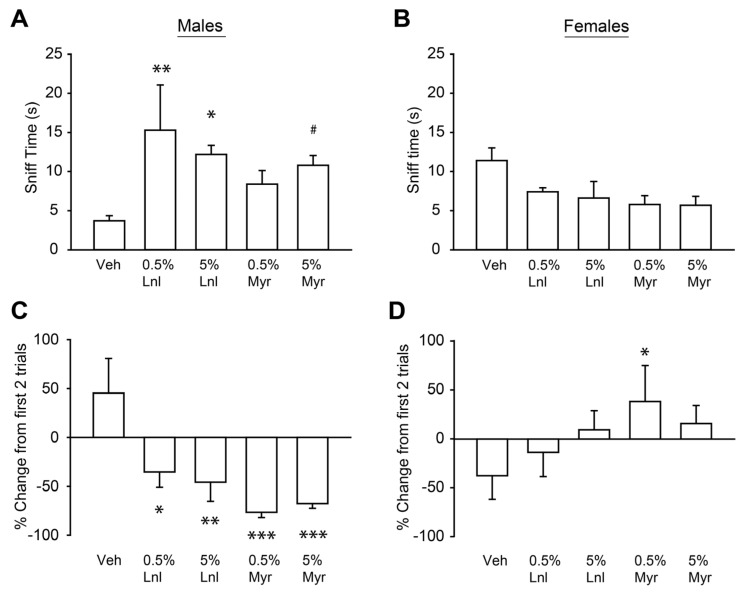
Sex differences in responses to repeated olfactory stimulation. (**A**) Summary bar chart showing the averaged sniff times in the first two trials of the olfactory detection and habituation procedure in males for the 5 different olfactory stimuli, n = 5. (**B**) Summary bar chart showing the averaged sniff times in the first two trials of the olfactory detection and habituation procedure in females for the 5 different olfactory stimuli, n = 5. (**C**) Summary bar chart showing the percent changes in the sniff time of each olfactory stimulus in the last two trials of the procedure compared to the first two trials for male mice. Note that negative change scores indicate reduced sniff time in the last two trials compared to the first two trials. (**D**) Summary bar chart showing the percent changes in the sniff time of each olfactory stimulus in the last two trials of the procedure compared to the first two trials for female mice. * indicates *p* = 0.05; ** *p* < 0.01; *** *p* < 0.001; # indicates *p* = 0.058. All statistical differences are shown compared to vehicle. Two-way mixed measures ANOVA assessed interactions between sex and exposure condition as well as any main effects. *p* values generated by Tukey’s HSD post hoc comparisons. Data are presented as mean ± SEM.

The sex differences we observed in the elevated plus maze and open field tests led us to hypothesize that males and females exhibited different sensitivities to the olfactory stimulus. We tested this hypothesis using an olfactory detection and habituation procedure in which sniff time was measured when mice were presented with a cue tip soaked in vehicle, 0.5% linalool or β-myrcene, or 5% linalool or β-myrcene for four 1 min trials per olfactory stimulus [43]. A two-way mixed measures ANOVA revealed that there was a significant interaction between sex and terpene condition on averaged sniff time in the first two trials, F(4,32) = 4.23, *p* < 0.01. We observed that males, but not females, engaged in significantly more sniffing time of the 0.5% linalool and 5% linalool-soaked cue tips compared to the vehicle (all *p* < 0.05; Figure 2A,B). Males also, on average, spent more time sniffing the 5% β-myrcene-soaked cue tip compared to vehicle-soaked one, but this effect did not reach our statistical significance threshold (*p* = 0.058). There were no significant differences between any of the terpene conditions among female mice across the first two trials. These findings suggest that male mice have the capacity to detect the terpenes, and further, it may be inferred from their increased sniff time that they do not find the terpenes aversive during short duration, acute exposure. We next measured the change in the averaged sniffing activity between the first two and last two exposures to see how the animals’ responses changed over repeated exposures that are similar to our vapor delivery methods. A two-way mixed measures ANOVA again revealed a significant interaction between sex and terpene condition on the change in sniffing behavior, F(4,32) = 9.61, *p* < 0.001. Compared to the change in sniffing behavior towards the vehicle-soaked cue tip, male mice showed significant reductions in sniffing behavior of all four terpene conditions in the last two trials compared to the first two trials (all *p* < 0.05; Figure 2C). Female mice increased their sniffing of the 0.5% β-myrcene-soaked tip compared to the vehicle (*p* < 0.05), but no other differences were observed (all *p*> 0.05; Figure 2D). These findings suggest that unlike males, females do not habituate to olfactory stimulation by these two terpenes. Together, we interpreted these findings to suggest that male mice were more sensitive than female mice to prolonged and repeated terpene exposure, which is consistent with our repeated vaporization protocol.

### 3.3. Short Terpene Exposure Has Anxiolytic Effects in Males

**Figure 3:**
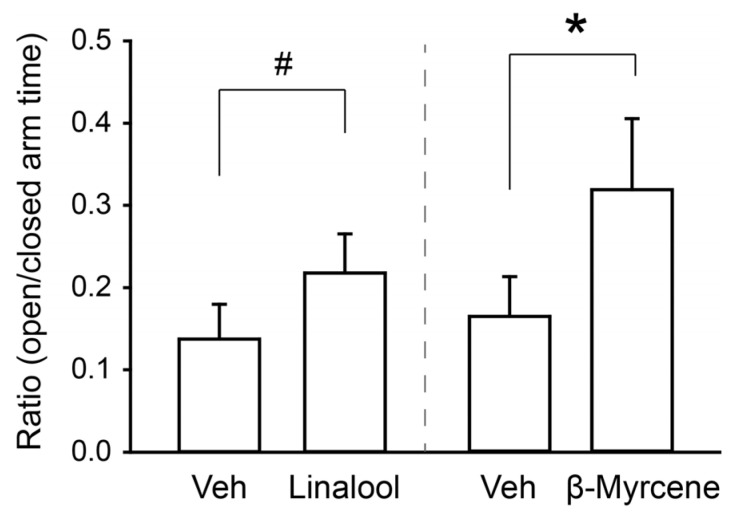
Short exposure to terpenes has anxiolytic effects in males. Summary bar chart showing the ratios of time males spent in the open relative to closed arms of the elevated plus maze following a single 3 s vapor pull of either vehicle (n = 5) or linalool (n = 5). * indicates *p* < 0.05; # indicates *p* = 0.58 by paired *t*-tests. Data are presented as mean ± SEM.

Based on the hypothesis that male mice were more sensitive to the olfactory stimulus and that the terpenes may still have anxiolytic potential in males if the exposure duration was reduced, we tested the effect of a single 3 s vapor pull of either linalool or β-myrcene in male mice. The exposure to this single “hit” lasted for approximately 1 min (58.40 ± 3.08 s) before the vapor was cleared from the chamber. Consistent with our hypothesis, paired *t*-tests revealed that β-myrcene increased the ratio of time spent in the open versus closed arms of the elevated plus maze, t(4) = 2.81, *p* < 0.05, but linalool’s effects did not quite reach our threshold for statistical significance, t(4) = 2.63, *p* = 0.058 (Figure 3). These findings indicate that that the intensity and duration of terpene exposure differentially impacts their anxiolytic properties in male and female mice. 

### 3.4. Anxiolytic Entourage Effects of CBD and Linalool in Female Mice

**Figure 4:**
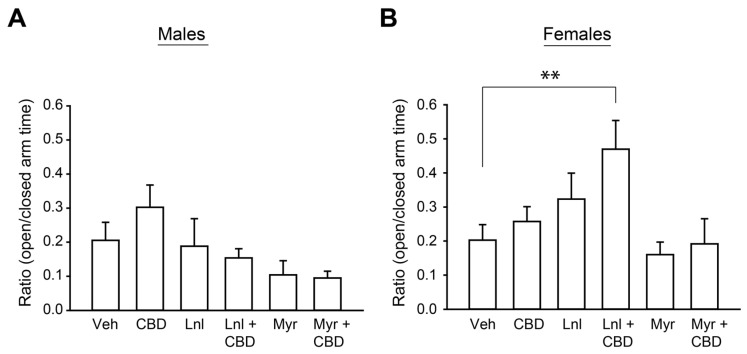
The combination of CBD and terpenes on elevated plus maze performance. Summary bar charts showing the ratios of time males (**A**) or females (**B**) spent in the open relative to closed arms of the elevated plus maze following 3 s pulls (note that this is half the pull length than shown in Figure 1) every 5 min for 30 min of vapor containing CBD or a terpene, alone or in combination. Two-way between-subjects ANOVA assessed interactions between sex and exposure condition. ** indicates *p* < 0.01 generated from Tukey’s HSD post hoc comparisons. Data are presented as mean ± SEM.

The Entourage Effect Hypothesis [47] posits that combining cannabinoids and terpenes enhances the therapeutic efficacy through either additive or synergistic interaction [13]. One potential outcome of this combinatorial effect that is predicted by the Entourage Effect Hypothesis is that sub-therapeutic doses of individual components become therapeutic when combined, for instance by lowering the therapeutic dosing widow. We tested this hypothesis by measuring the effect of linalool or β-myrcene, alone or in combination, with CBD (30 mg/mL) at half the terpene’s therapeutic pull duration we had previously identified on the elevated plus maze (i.e., 3 s vapor pulls instead of 6 s pulls). A two-way between-subjects ANOVA revealed that neither of the terpenes nor CBD at this dosing level individually reached the criteria for anxiolytic effectiveness. However, we found a significant interaction between sex and treatment condition on the ratio of time spent in the open versus closed arms in the elevated plus maze, F(5,113) = 2.36, *p* = 0.04. In female mice, the combination of CBD + linalool increased the time spent in the open versus closed arm compared to vehicle treatment (*p* < 0.05). The enhanced anxiolytic effect from the addition of CBD to linalool was not observed with the addition of CBD to β-myrcene (*p* > 0.05). None of the exposure conditions improved the open/closed ratio in male mice (all *p* > 0.05; Figure 4A,B). These findings suggest that there may be sex-dependent sensitivities to the combinatorial actions of CBD with terpenes and that the enhanced therapeutic benefits achieved with these combinations are terpene dependent for each therapeutic purpose.

## 4. Discussion

### 4.1. Exposure Pattern of Terpene Delivery Impacts Anxiolytic Effects in a Sex-Dependent Manner

Anxiety and anxiety-related complications are the most common reason for off-label medicinal hemp use [11,48,49]. While empirical demonstrations of CBD’s anxiolytic effects are becoming more common [32,50], there is little empirical understanding of independent contributions that prominent monoterpenes or monoterpenoids, found in whole-plant cannabis extracts, contribute to the plant’s purported anxiolytic effects. In this study, we demonstrated that vapor delivery of a prominent monoterpenoid and monoterpene, linalool and β-myrcene, respectively, which are found in commercially-available cannabis products [22], have anxiolytic effects in mice assessed using the elevated plus maze assay. However, the exposure characteristics that promote these anxiolytic effects differ as a function of mouse sex: anxiolytic effects are observed in female mice following repeated and prolonged exposure whereas anxiolytic effects are only observed in males following a short, acute exposure. Furthermore, we found that combining sub-anxiolytic levels of linalool and CBD leads to substantial anxiolytic effects in female mice. These findings reveal sex-dependent differences, which if they exist in humans, could impact the anxiolytic potential of cannabis products and inform efficacious consumption strategies. 

### 4.2. Sex Differences in Olfactory-Based Behaviors

Sex differences in the effects of olfactory stimulation from essential oils are not always assessed in rodent studies, which traditionally have utilized male animals, but there is some evidence that sex differences exist in mice [51], rats [52], and gerbils [53] when both sexes are included. The sex-dependent response profile differs based on the essential oil and show differential effects on anxiety [53], pain [52], and neurotransmitter release [54] that emerge after prolonged exposure periods from several minutes up to 2 weeks. Our findings add to this growing body of evidence for sex differences in olfactory stimulation to linalool and β-myrcene on locomotor activity and anxiety. 

Sex differences in odorant effects have also been observed in humans. One study found that diffusion aromatherapy with linalool-rich lavender essential oil reduced anxiety and elevated oxytocin in women, whereas there was no clear relationship between aromatherapy, anxiety, or oxytocin levels in men [55]. Similarly, essential oil of orange diffused into the waiting room of a dental office reduced anxiety and improved mood in females but not males [20]. Another study found that olfactory stimulation with peppermint increased non-REM sleep in females but not males. Conversely, peppermint increased alertness in males but not females [56]. These experiments used ambient diffusion techniques that lead to consistent olfactory stimulation. Although we exposed mice to discrete vapor pulls over the course of a half hour, our findings that male mice did not respond as positively to olfactory stimulation as females with repeated exposures is consistent with the findings from these diffusion experiments in humans. 

In some cases, a lack of terpene effects may indicate that desensitization has occurred. In the zebrafish model, the effects that acute exposure to β-myrcene had on locomotor activity went away following several days of consistent exposure, which was interpreted as desensitization to prolonged terpene effects [57]. However, in our research, it is unlikely that we were detecting desensitization in males and instead, may have been observing the emergence of aversive qualities that may result from being overwhelmed from extensive repeated exposure to the terpene. We found that six vapor pulls of linalool over a 30 min period increased anxiety-like behavior on the elevated plus maze and open field test in males compared to vapor exposure to the vehicle solution. Similarly, while males increased their sniff time of the vehicle solution in the last two trials of the olfactory habituation procedure compared to the first two trials, they drastically reduced their sniff time of linalool and myrcene odors. This is consistent with a much more prolonged exposure study where 2 weeks of consistent exposure to the essential oil of citrus lemon enhanced anxiety-like activity in the elevated plus maze [52]. Together, these findings suggest that over-exposure to terpenes may impede their hedonic and anxiolytic effects, although future studies should directly investigate the hedonic or anhedonic characteristics of terpenes, since this knowledge could inform effective consumption characteristics or product development. 

### 4.3. Potential Mechanisms Underlying Sex Differences in Olfactory Sensitivity

The molecular mechanisms behind our observed sex differences in behavioral responses to terpenes are uncertain. There are several factors that promote sexual dimorphic olfactory processing. For instance, olfactory receptor genes are highly dimorphic and lead to altered chemoreceptor expression that may lead to differential response phenotypes for odorants [58]. Experience can alter olfactory neuron expression, in part through neuroplasticity induced by mitral cells in the olfactory bulb [59], and populations of mitral cells are developmentally regulated by sex steroids [60]. Therefore, sex hormones can impact relevant olfactory circuits that could differentially impact the magnitude of terpene-activated signaling between the sexes. Furthermore, there may be sex differences in central targets of these terpenes beyond the olfactory epithelium. For instance, intraperitoneal injection of linalool caused hypolocomotion in CD-1 mice that was mediated by CB1 receptors in males and adenosine A2a receptors in females [61]. Together, these studies propose that that sex-dependent variation in molecular targets at the level of the olfactory receptor neurons, sex steroid-dependent plasticity of centrally-projecting olfactory neurons, and pharmacological brain targets, could underlie sex differences in the sensitivity to common cannabis terpenes. Understanding the mechanism(s) underlying sex differences in terpene sensitivity and its effects is important for optimizing terpene-based therapeutic strategies and delivery systems to achieve more reliable outcomes. 

### 4.4. Entourage Effects of CBD and Linalool on Anxiety-Related Behavior

Another main finding is that the combination of sub-effective levels of CBD and linalool enhanced the anxiolytic effectiveness in female mice. This findings supports the Entourage Effect Hypothesis, which posits that inactive or sub-efficacious compounds can enhance the effect of cannabinoids [62]. Based on this hypothesis, the combination of phytocannabinoids (e.g., CBD) and terpenes (e.g., linalool) would be more efficacious than the phytocannabinoids on their own [47]. Indeed, we observed evidence of the entourage effect with linalool in female mice. Initially, we found that 6 s vapor pulls of linalool delivered every 5 min for 30 min had anxiolytic effects in females. Linalool did not produce anxiolytic effects when the vapor hit duration was cut in half to 3 s pulls, nor did CBD have anxiolytic effects at this hit duration. The combination of the two, however, did reach our statistical threshold for anxiolytic behavior. This supports the hypothetical but previously untested assertion that the combination of linalool and CBD would lead to more robust anxiolytic effects by expanding the effective dose range of the primary phytocannabinoid. Since one of the major hurdles with using CBD to reduce anxiety is its narrow effective dose range [33,63,64,65], the addition of linalool may expand this dose range and lead to more predictable and effective use. 

We did not observe this entourage effect with the combination of CBD and β-myrcene for anxiety-related behavior. We present the first known evidence that β-myrcene, as a monoterpene, has anxiolytic effects in female mice. However, when exposure levels are reduced to a sub-effective level, we do not find that the addition of low amounts of CBD has any additive or synergistic anxiolytic value. The different pharmacodynamic profiles of β-myrcene and linalool may contribute to the variation in effects when combined with CBD [13]. Since many of the prominent cannabis terpenes do not potentiate cannabinoid activity directly at cannabinoid receptors [66], there is substantial potential for synergistic action between terpenes and CBD through indirect action on endocannabinoid function or independent of the endocannabinoid system altogether [23,67,68,69]. 

### 4.5. Potential Mechanism Underlying Myrcene and Linalool’s Anxiolytic Effects 

The exact mechanism of β-myrcene and linalool’s anxiolytic action requires further investigation. We observed that 30 min of repeated vapor pulls of linalool enhanced locomotor activity in males, but β-myrcene enhanced it in females, despite anxiolytic effects of both terpenes using this exposure protocol in females only. This suggests a dissociation between the underlying neural substrates of these terpenes that affect locomotor activity and anxiety-related behavior. Nonetheless, both terpenes are shown to modulate neurotransmitter levels through direct or allosteric [70] modulation of receptors or channels that could contribute to sedative or anxiolytic action [70,71,72,73]. However, it is unknown if these terpenes accumulate to reach brain levels from our exposure protocol sufficient to induce direct changes on central neurons. Instead, the observed anxiolytic and locomotor effects may have solely been the result of olfactory stimulation. Consistent with an olfactory-dependent mechanism of action, Harada et al. [27] found that linalool’s anxiolytic effect in male mice was absent in anosmic mice whose olfactory epithelia had been ablated. Notably, they did not assess the necessity of olfactory stimulation for linalool’s anxiolytic effects in females. Future studies should investigate whether differences in the anxiolytic characteristics of β-myrcene and linalool between males and females derive from differences in olfactory stimulation and other neural targets. Not only will this inform effective use strategies but could also lead to improved delivery mechanisms that seek to optimize the delivery of cannabinoids and terpenes via therapeutically relevant pathways based on a person’s unique demographics. 

### 4.6. Limitations of the Current Study

There are several limitations of this study. One notable caveat is that we observed variability across baseline EPM performance in vehicle-treated male mice. Although we sought to control for this using a counterbalanced within-subjects design for assessing treatment effects shown in EPM-related Figure 1 and Figure 3, the inherent variability warrants replication to demonstrate the consistency of antipodal responses to repeated terpene exposure between males and females. An additional limitation is that we only tested anxiety behavior using the elevated plus maze. There are additional assays for testing anxiety, such as the light/dark box, that could also be employed to test the effect of these terpenes on anxiety-related behavior. Time in the center quadrant of the open field chamber is sometimes used as a proxy for anxiety, although we only found that repeated linalool stimulation decreased time in the center quadrant in male mice. Future studies should assess anxiety using additional assays to ensure that the effects observed here are not limited to a particular assay. Additionally, future studies should include a non-anxiolytic terpene to serve as a negative control. Although our vehicle comprising vegetable glycerin and propylene glycol has an odor (see Figure 2) and the terpenes assessed have differential effects when combined with CBD at sub-therapeutic levels (see Figure 4), a control terpene without anxiolytic effects would provide a stronger argument that the behavioral effects we observed are due to the terpenes’ effects on anxiety-related behaviors and not merely olfactory novelty. Since olfactory novelty can boost exploratory behavior, especially in the C57BL/6J mice studied here [74], stronger olfactory signaling by any terpene could contribute to an enhanced behavioral phenotype on the EPM. It is currently unclear how the differing olfactory sensitivities to the tested terpenes across sexes would generalize to a range of terpenes, and this warrants testing. Another limitation is that we tested a limited exposure range of terpenes and CBD. We have previously demonstrated a dose-dependent anxiolytic effect of CBD in the BTBR mouse model of autism spectrum disorder [41], but the dose-dependent relationship of the terpenes on anxiolytic behavior (i.e., manipulating concentration in oil, not exposure time) has yet to be tested. Therefore, it is unclear if terpene concentration or exposure duration has the greatest impact over anxiety-related behavior. Consequently, it is feasible that we missed potential entourage effects between β-myrcene and CBD as we observed for linalool and CBD, by not fully assessing a wider range across these parameters. We also cannot ensure consistent inhalation and exposure levels between animals and from trial to trial. There is notable variability in blood drug levels following the vapor delivery method [75], and so we sacrificed dose control for modeling valid cannabis inhalation patterns. We feel this vaporization method is a strength of the current study since it best mimics human use patterns where cannabinoids and terpenes are consumed through acute bouts of vaporized oils or combusted flower. However, future studies should seek to correlate behavioral outcomes with plasma or brain concentrations of terpenes. Given the importance of puff frequency and total exposure duration on β-myrcene and linalool’s effects, it is important for future studies to model human use patterns that, in this case, best relate to cannabis consumption as opposed to constant diffusion found with aromatherapy. 

## 5. Conclusions

We report that (1) there are sex differences in the exposure durations to vaporized linalool and β-myrcene that promote anxiolytic effects on the elevated plus maze, and (2) the addition of CBD to linalool can enhance its anxiolytic potential. These outcomes were observed using vaporization exposure methods common among cannabis users who combust follower or vape cannabis oils and at relevant terpene concentrations found in commercially available strains and products [22]. Based on these findings, users should be aware that different puff patterns may impact their subjective effects. Future studies should address the mechanisms underlying sex differences in the response to terpenes to improve effective use strategies. 

## Data Availability

The data presented in this study are available on request and without reservation from the corresponding author.

## Supplementary Materials

The following supporting information can be downloaded at: https://www.mdpi.com/article/10.3390/neurosci5040045/s1, Table S1: Descriptive statistics.
